# Pulmonary tuberculosis in an immunocompetent patient with primary laryngeal aspergillosis

**DOI:** 10.1002/rcr2.586

**Published:** 2020-05-17

**Authors:** Nirmal Kanti Sarkar, Bijoy Pada Gope

**Affiliations:** ^1^ Department of Respiratory Medicine Mugda Medical College Dhaka Bangladesh; ^2^ Department of Respiratory Medicine M.A.G. Osmani Medical College Sylhet Bangladesh

**Keywords:** Aspergillosis, healthcare worker, larynx, pulmonary tuberculosis

## Abstract

Primary laryngeal aspergillosis in an immunocompetent host is a rare entity. On the other hand, pulmonary tuberculosis (PTB) among healthcare workers in a tuberculosis endemic zone with high risk of exposure is not uncommon but may be underdiagnosed especially when masquerading as another disease. In this report, we are presenting a 45‐year‐old physician who presented with chronic dry cough, hoarseness of voice, and progressive vocal fatigue. Fibreoptic laryngoscopy (FOL) showed whitish patches on both vocal folds and he was initially diagnosed with laryngeal aspergillosis following histopathological examination. As there was no significant improvement on antifungal treatment, we re‐evaluated the case and, on further investigation, concomitant PTB was detected. Patient responded to category‐I anti‐tubercular drugs with complete recovery.

## Graphical abstract

A 45‐year‐old physician presented with dry cough and progressive vocal fatigue and was diagnosed as primary laryngeal aspergillosis. Further work‐up revealed coexistent pulmonary tuberculosis.

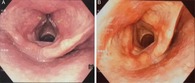

## Introduction

The larynx is an unusual site for *Aspergillus* colonization, which mostly occurs in advanced stages of bronchopulmonary infection in immunocompromised hosts. Aspergillosis primarily involving the larynx in an immunocompetent individual is very rare [1]. Cough and voice change are the presenting features [2]. Coexisting pulmonary tuberculosis (PTB) in the same patient makes the scenario a complex one. Here, we report a case where the patient was initially diagnosed with primary laryngeal aspergillosis and, on further evaluation, concomitant PTB was detected.

## Case Report

**Figure 1:**
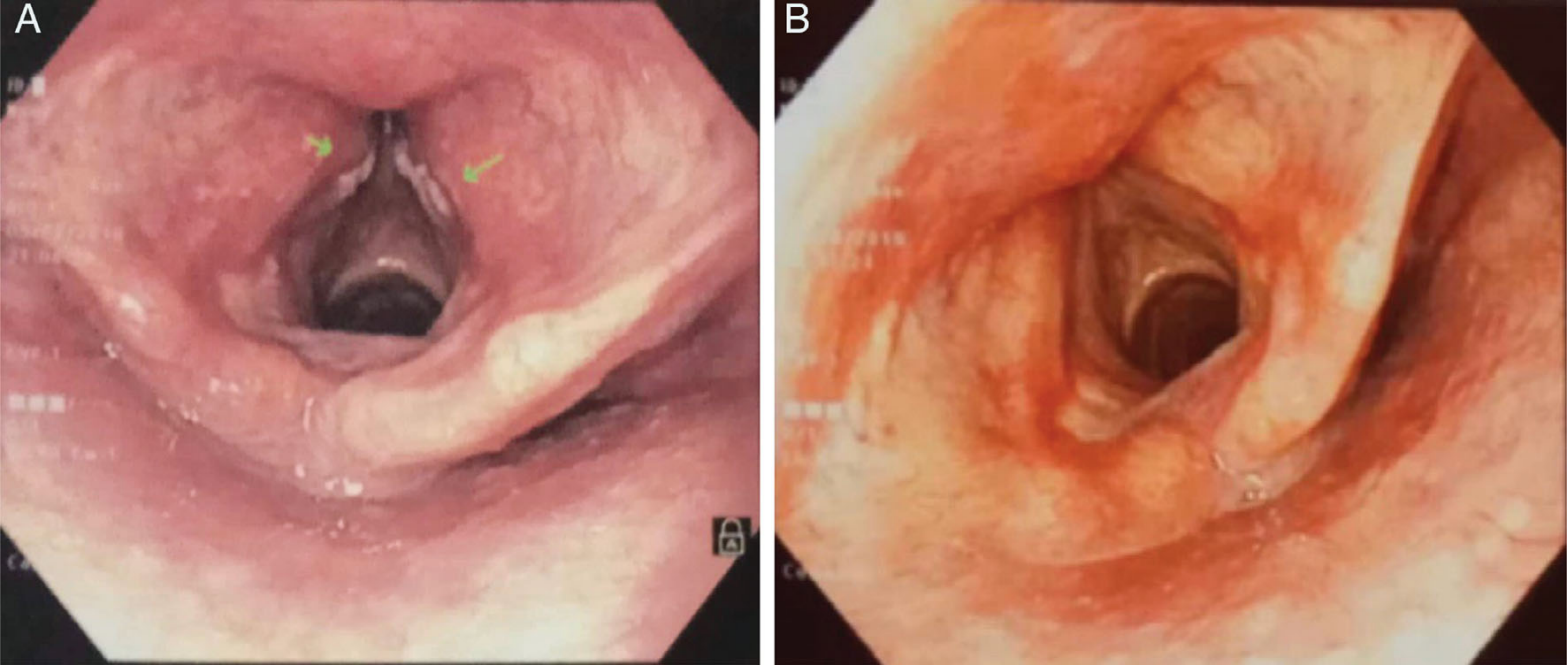
Fibreoptic laryngoscopy showing whitish patches (arrows) on anterior part of both vocal folds (A) and follow‐up after one year (B).

**Figure 2:**
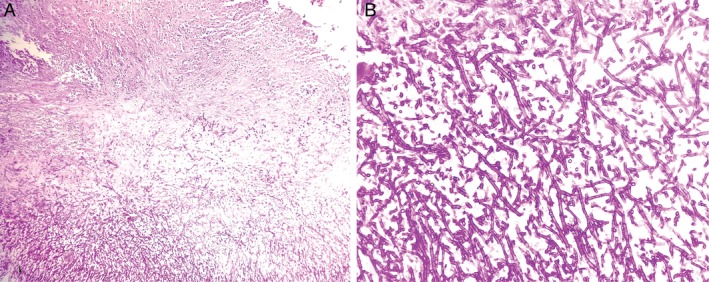
Histopathology of biopsy specimen from the right vocal cord showing ulcerated squamous epithelium and fungal hyphae [Periodic Acid‐Schiff (PAS), 100×] (A) and branching septate hyphae of *Aspergillus* spp. (PAS, 400×) (B).

A 45‐year‐old man presented with dry cough and progressive vocal fatigue for two months and fever for ten days. Following seven days of treatment with oral antibiotic, prednisolone, montelukast, inhaled corticosteroid (ICS), and inhaled bronchodilator, his symptoms were partially relieved but he developed hoarseness of voice a few days later. A chest X‐ray was normal. Due to persistent symptoms, he consulted an otorhinolaryngologist and was referred for fibreoptic laryngoscopy (FOL). FOL showed whitish patches over anterior third of both vocal folds (Fig. 1A). Fungal laryngitis was suspected and he was prescribed oral itraconazole. Despite getting three weeks of treatment, vocal fatigue and cough persisted. Repeat FOL was done and biopsy was taken under general anaesthesia. Histopathology revealed ulceration of vocal cord mucosa lined by granulation and necrosed tissue. Numerous hyphae of *Aspergillus* were found in necrosed tissue (Fig. 2). No granuloma or malignant cell was seen. Fungal culture was not done. Oral voriconazole was started 200 mg 12 hourly. After two weeks of treatment, hoarseness of voice partially improved but cough and low‐grade fever persisted and he developed hepatitis.

**Figure 3:**
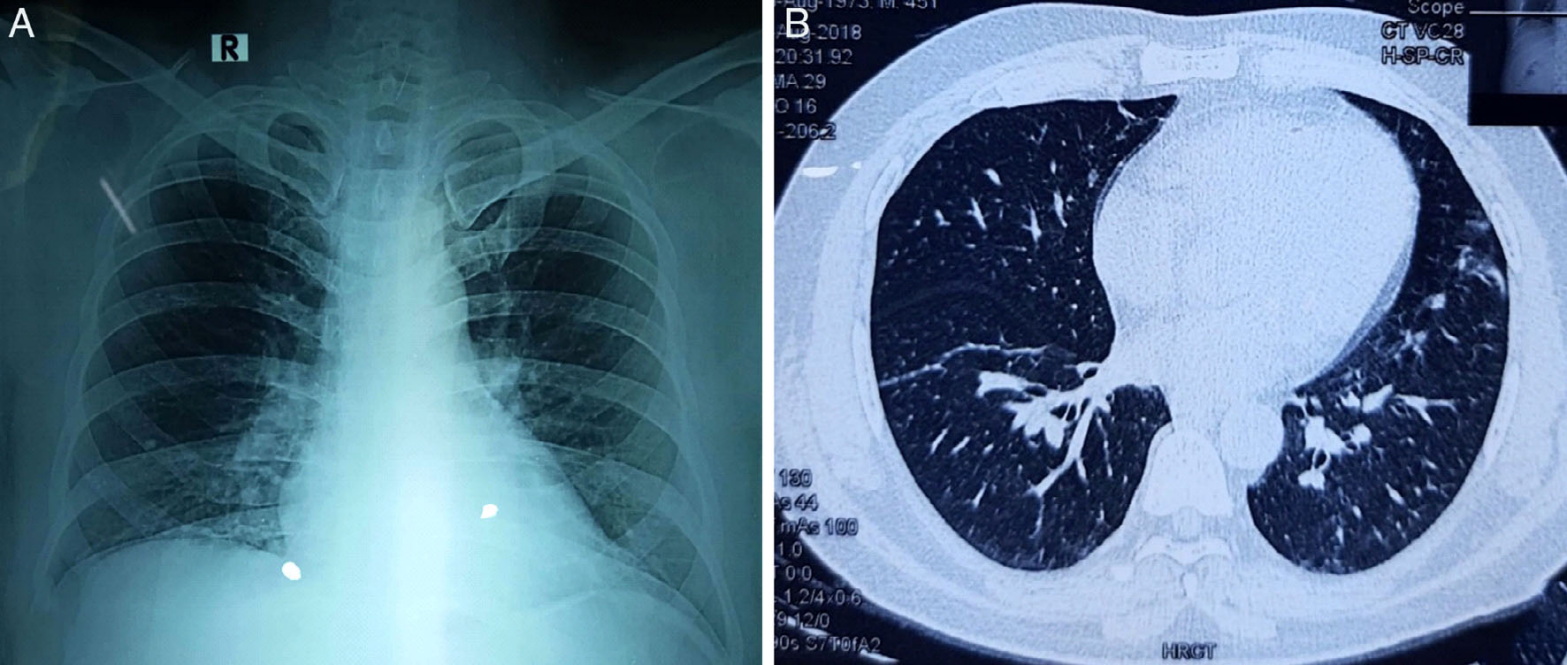
Normal chest X‐ray (A) and high‐resolution computed tomography (HRCT) scan (B).

He is a physician and was working in a chest disease clinic with frequent exposure to tuberculosis (TB) patients. There was no history of diabetes mellitus, malignancy, or immunodeficiency. HIV serology was negative. He is non‐smoker, had allergic rhinitis, mild persistent asthma, and took low‐dose inhaled fluticasone (500 μg/day) irregularly. On examination, he looked ill and chest examination revealed normal findings. Chest X‐ray and computed tomography (CT) scan were unremarkable (Fig. 3). Sputum acid fast bacilli (AFB) and GeneXpert (Cepheid, Inc., Germany) were negative, and erythrocyte sedimentation rate (ESR) was 84 mm in the first hour, serum bilirubin 0.45 mg/dL, and serum glutamate pyruvate transaminase (SGPT) was 242 U/L. Keeping in mind about the patient's occupation, and as he had frequent occupational exposure to smear‐positive TB patients without adequate personal protection, fibreoptic bronchoscopy and study of bronchoalveolar lavage (BAL) were planned. Fibreoptic bronchoscopy (FOB) revealed mucosal hyperaemia of right lower lobe bronchus. Bronchial lavage was taken from the right lower lobe. *Mycobacterium tuberculosis* was detected in BAL GeneXpert with rifampicin sensitive. The final diagnosis of PTB with primary laryngeal aspergillosis was made. Treatment with category‐I anti‐TB drug was started after resolving hepatitis and he was advised to stop ICS and practice prevention measures at the workplace. He took voriconazole for a total of four weeks. His condition gradually improved with resolution of cough, fever, vocal fatigue, and hoarseness of voice. Follow‐up bronchoscopy done one year later showing complete resolution of vocal cord lesion (Fig. 1B).

## Discussion

Aspergillosis is a fungal infection caused by the genus *Aspergillus*, having very little pathogenic capability in a healthy host. The organism gains access to the respiratory mucosa via inhalation of spores with subsequent invasion causing necrosis, ulceration, haemorrhage, and thrombosis. The larynx is an unusual site of *Aspergillus* infection, which may be involved in advanced stage of bronchopulmonary aspergillosis in an immunosuppressed individual.

Primary laryngeal aspergillosis in immunocompetent individual is extremely rare and often mimics premalignant or malignant condition of the larynx. Globally, only 38 cases have been documented over the last five decades [2]. Patients commonly present with sore throat, hoarseness of voice, cough, and odynophagia [3], and our patient had progressive vocal fatigue. The pathogenesis of laryngeal aspergillosis is not well understood but is most likely multifactorial. Several factors may be responsible for the development of disease which include ICS, vocal abuse, smoking, laryngeal radiation, prolonged systemic and nebulized antibiotic, vocal cord cyst, and oral sex [2]. A study by Dutta et al. found that 50% of immunocompetent subjects with primary laryngeal aspergillosis had no identifiable factors [3]. Several studies showed that local factors are responsible for isolated laryngeal aspergillosis rather than immunosuppression.

Our patient had a history of using occasional ICS for bronchial asthma. A substantial portion of steroid may be deposited in the larynx, leading to a breach in local immunity of the throat, alteration of the normal flora of the larynx, or cause erosion of the laryngeal mucosa, allowing *Aspergillus* colonization [4]. Although our patient used ICS irregularly in low dose, studies showed that even lower dose ICS may lead to favourable condition of fungal colonization in larynx [4]. We also observed that our patient did not respond to initial treatment with itraconazole. In the majority of reported cases, itraconazole was used empirically for laryngeal aspergillosis, but voriconazole is the drug of choice against invasive aspergillosis [2]. As the patient was not improving on antifungal treatment, we sought for any associated disease. Persistent cough, low‐grade fever, and his occupation with frequent exposure to smear‐positive TB patients compelled us to exclude TB. Bangladesh is one of the countries of highest TB burden. According to the World Health Organization (WHO) Global Tuberculosis Report 2019, the incidence rate of TB in Bangladesh was 221/100,000 population in 2018. An earlier study has shown that the prevalence rate of new TB cases in Dhaka City was 253/100,000 population [5]. Globally, healthcare workers (HCWs) are at high risk of latent TB infection or active TB disease through occupational exposure to active TB patients. A recent meta‐analysis showed that the prevalence of latent TB infection among HCWs was 37% and the mean incidence rate of active TB was 97/100,000 per year [6]. So our suspicion was justified, although it is difficult to comment which disease he acquired first. Lee et al. in their study in South Korea observed that ICS may increase the risk of TB by decreasing local immunity of the lung or by reactivating latent TB [7]. So, it is very much possible that ICS may have had a crucial role in the development of both diseases in our patient.

Our patient is unique in perspective of harbouring two diseases—primary laryngeal aspergillosis and PTB. To the best of our knowledge, this is the first reported case having both diseases in the same patient. Primary laryngeal aspergillosis in immunocompetent patient is an emerging trend and HCWs are at increased risk of TB infection. This curious case had dual pathologies. Rational suspicion leads us to a definite diagnosis and favourable outcome.

### Disclosure Statement

Appropriate written informed consent was obtained for publication of this case report and accompanying images.
